# Object-Based Paddy Rice Mapping Using HJ-1A/B Data and Temporal Features Extracted from Time Series MODIS NDVI Data

**DOI:** 10.3390/s17010010

**Published:** 2016-12-22

**Authors:** Mrinal Singha, Bingfang Wu, Miao Zhang

**Affiliations:** 1 University of Chinese Academy of Sciences, Beijing 100049, China; msingha@radi.ac.cn; 2 Division of Digital Agriculture, Institute of Remote Sensing and Digital Earth, Olympic Village Science Park, Beijing 100101, China; zhangmiao@radi.ac.cn

**Keywords:** paddy rice mapping, object-based, fusion, classification, HJ-1A/B, temporal features, Assam

## Abstract

Accurate and timely mapping of paddy rice is vital for food security and environmental sustainability. This study evaluates the utility of temporal features extracted from coarse resolution data for object-based paddy rice classification of fine resolution data. The coarse resolution vegetation index data is first fused with the fine resolution data to generate the time series fine resolution data. Temporal features are extracted from the fused data and added with the multi-spectral data to improve the classification accuracy. Temporal features provided the crop growth information, while multi-spectral data provided the pattern variation of paddy rice. The achieved overall classification accuracy and kappa coefficient were 84.37% and 0.68, respectively. The results indicate that the use of temporal features improved the overall classification accuracy of a single-date multi-spectral image by 18.75% from 65.62% to 84.37%. The minimum sensitivity (MS) of the paddy rice classification has also been improved. The comparison showed that the mapped paddy area was analogous to the agricultural statistics at the district level. This work also highlighted the importance of feature selection to achieve higher classification accuracies. These results demonstrate the potential of the combined use of temporal and spectral features for accurate paddy rice classification.

## 1. Introduction

Rice is the principal food for nearly 50% of the world’s seven billion people, mostly in Asia, Africa and Latin America [1]. Paddy rice covers more than 12% of global cropland areas [2], and it consumes about 24%–30% of the world’s developed fresh water resources [3]. Rice is the largest water-consuming crop and is cultivated primarily in constantly flooded fields. Therefore, future expansion of the fields may lead to water shortage and ecosystem disturbance [3]. Rice cultivation can contribute to climate change [4] as the flooded paddy fields are responsible for 10% of human-induced methane (CH_4_) [5], or 20% of total agricultural CH_4_ emissions [5]. Paddy fields have also been identified as a transmission medium of highly pathogenic avian influenza virus H5N1 [6]. Increasing urbanization, rising global temperature, industrialization, and changing precipitation patterns are affecting the land and water resources of rice production [7]. Therefore, it is important to monitor and map the paddy rice fields for the assessment of food security, efficient water resources management, environmental sustainability, and controlling the transmission of influenza viruses. 

Remote sensing has been proved to be an effective tool for mapping the paddy rice fields, employing easily available optical and synthetic aperture radar (SAR) images [8,9,10,11,12]. Recently, significant efforts have been carried out towards the mapping of paddy rice using the time series data [13,14,15,16]. A variety of techniques have been used for paddy rice mapping such as supervised classification [17,18], a thresholding based method [19,20,21], phenology based mapping [13,22] and a subtraction based method [23,24]. However, a number of limitations exist with these methods. For example, supervised classification requires training samples for each year, and incorrect samples often lead to unexpected results. Thresholding requires appropriate selection of threshold values and subjectivity remains in the classification results due to the intra-class variability. Phenology based mapping needs continuous and long time series, which is a challenge for a large area. A subtraction based method mandatorily requires a shortwave infrared band (SWIR) to calculate land surface water index (LSWI). However, SWIR is not available in many satellite sensors such as WorldView-2, Chinese HJ-1A/B, ZY-3 MUX and GF-1 WFV. Additionally, all of these methods underperform where the rice fields are fragmented. 

Earlier studies have revealed the importance of considering pixel-level spectral heterogeneity and parcel sizes to the classification processes [25]. However, traditional pixel-based classification methods are unable to incorporate this information, which limits their application mainly in the regions where crop fields are fragmented with high spectral variability. Object-based image analysis (OBIA) can address these issues considering the spatial location and context of homogenous pixels [26]. Segmentation, the initial step in OBIA, is the grouping of pixels to form the internally uniform homogeneous objects [27]. These objects generate textural and geometric information in addition to spectral information, which helps conduct more efficient and improved classification [28,29]. This approach is particularly useful where the crop fields are fragmented and relatively small in size.

Fine resolution paddy rice maps are usually generated from a single or a few images obtained during growing season. Recently, researchers have been attracted by the fine resolution mapping of paddy rice, especially using the time series Landsat data [15,16,23]. The temporal or phenological information contained in the time series has been obviously useful for paddy rice mapping [30]. However, the requirement of intensive data processing makes these approaches limited in small regions. Additionally, due to the frequent cloud cover and long revisit cycle (16-days for Landsat), it is difficult to obtain a dense time series. Thus, rice mapping using a time series is usually focused on coarser resolution data [21,31]. The combined use of fine and coarse resolution images potentially increases the data availability for better tracking of crop phenology and efficient paddy rice mapping. Therefore, it has a great potential to improve paddy rice classification if temporal features of coarse resolution data are used in classifying finer resolution data. 

The main challenge is how to extract the temporal features from time series coarser resolution data to be used for fine resolution spectral features for improved classification. Blending or fusion of coarse and fine spatial resolution images have been proved to be a feasible solution for the issue [32,33]. Many studies utilized fused time series vegetation index datasets for cropland mapping and phenology studies [34,35]. However, fused datasets are rarely utilized to improve paddy rice classification of fine resolution data. The main objective of this study is to evaluate the potential of temporal features extracted from fused time series datasets for OBIA based paddy rice classification.

## 2. Study Area

**Figure 1:**
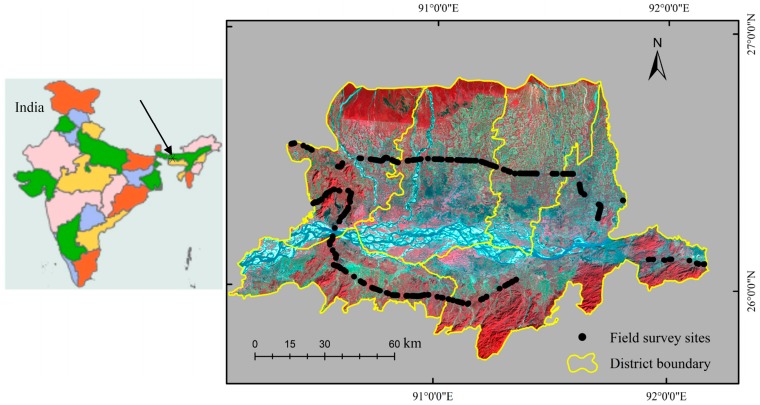
Location map of study site in Assam, India. The background image is a HJ-1A false color composite (**red**: NIR band, **green**: Red band, **blue**: Green band) of 5 December 2014.

The study area is located in northeast India, centered at 26°23’ N and 91°09’ E (Figure 1). The selected site includes five districts of Assam state with an area about 14,000 km^2^. The region is a flood plain of the Brahmaputra and Barak rivers. It has a tropical monsoon climate with high rainfall and humidity. The average annual precipitation and the temperature are approximately 3000 mm and 23 °C, respectively. Rice, as the primary crop of the region, is cultivated not only in the plains but also in the hill slopes. Rice is occupying 25 million hectares in the region, which accounts for 71% of the total cultivated area [36]. Based on the usage of water, rice is cultivated in a variety of environmental conditions in the region. The most common cultivation types include: (a) rainfed lowland; (b) irrigated; (c) flood-prone and (d) upland. The region has primarily two rice growing periods: rabi or summer (March–July) and kharif or winter (June–December). In kharif season, rice is cultivated widely due to the abundant rain and the favorable temperature. During rabi season, rice is cultivated in the areas where the irrigation facilities are available. This study considers only the kharif season paddy rice for the mapping.

## 3. Dataset and Pre-Processing

### 3.1. MODIS NDVI Data

**Figure 2:**
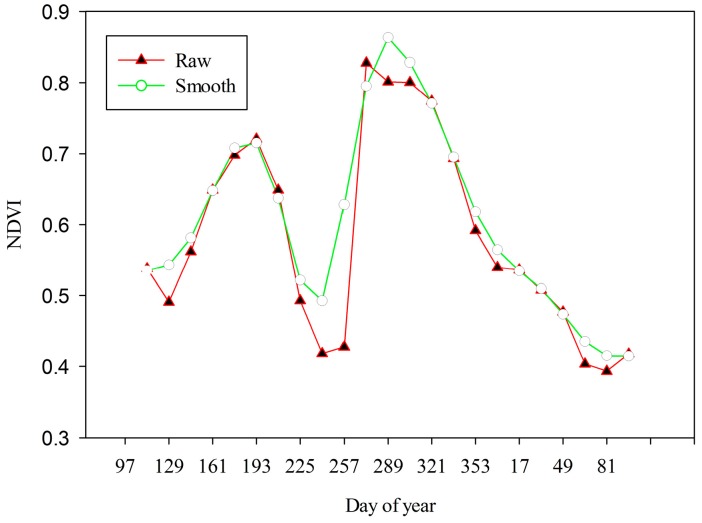
Raw and smoothed time series NDVI for a randomly selected pixel of an agriculture landscape.

The Moderate resolution imaging spectroradiometer (MODIS) (MOD13Q1, Collection 5) Normalized difference vegetation index (NDVI) dataset covering the study area from April 2014–March 2015 (Table 1) were acquired from the Land Processes Distributed Active Achieve Center (LP DAAC) [37]. The dataset is a 16-day composite and 250-m spatial resolution. The time series MODIS NDVI contains a significant amount of noise due to cloud contamination, aerosol and atmospheric effect [38]. The Savitzky–Golay (S–G) smoothing filter [38] was applied to remove the noise from the time series MODIS NDVI data. It is essential to remove the noise for the further use of the time series, particularly for the accurate extraction of temporal features [39]. An image based S–G filter was implemented in the IDL programming language for the purpose. The S–G filter smoothed the data by applying a locally adaptive moving window with a polynomial least square regression fit for approximating the noisy data. The filter well preserved the heights and widths of time series curve. In Figure 2, the original and smoothed NDVI are shown for an agriculture landscape. Finally, the smoothed dataset was re-projected to UTM, Zone 46N and resampled at 30-m spatial resolution by the Nearest Neighbor method. The resampled dataset was further used as an input to the image fusion process. 

### 3.2. HJ-1A/B Data 

The Chinese HJ-1A/B is a sun-synchronous earth observation satellite on board with two Charge-coupled device (CCD) sensors. The sensors acquire the data with 30-m spatial resolution at nadir angle using four spectral bands ranging from visible to near-infra red wavelengths. The satellite provides rapid coverage of grounds with a four-day repeat cycle. The dataset was downloaded from the China Center for Resources Satellite Data and Application, CRESDA [40] (Table 1). The downloaded dataset was of good quality with less than 10% cloud cover. The technical specification of the dataset is provided in Table 2. The following pre-processing procedures were performed on the datasets: (a) radiometric calibration using the calibration coefficient provided by CRESDA [40]; (b) atmospheric correction using the FLAASH model available at ENVI; (c) geometric correction; (d) removing of bad observations including thin clouds and shadows by using the modified neighborhood similar pixel interpolation (NSPI) approach [41]; and (e) mosaicking and clipping in the extent of the study area.

### 3.3. Field Data

To assist the accuracy assessment of the paddy rice map, an extensive field survey was carried out in May 2015. A detailed survey route map was designed according to the recent road maps as available in the fine resolution Bing images [42]. The data was collected using a GPS-video-GIS instrument (GVG) developed by Wu and Li [43]. The GVG instrument was installed on a vehicle travelling along the designed routes. The camera integrated with the GVG snaps photos at a specified time interval and the GPS receiver records the geo-location of each photo [43]. A distance of about 400 km was travelled along the designed routes collecting more than 1800 ground points (see Figure 1). Each photo collected by the GVG survey was visually interpreted using the GVG visual interpretation Graphical User Interface (GUI) and assigned respective land use land cover (LULC) classes to the photos. The collected photos include the paddy fields and major LULC classes. All of the field photographs will be shared to the user community by the ‘Global Geo-Referenced Field Photo Library’ a web-based data portal open to the public, researchers and stakeholders [44]. 

### 3.4. Ground Reference Data Generation 

A ground reference map was created by analyzing the following auxiliary data sets: (a) LULC of 2013–2014 at the scale of 1:250,000 and 1:10,000 available at the Bhuvan geo-portal [45]; (b) field surveyed data of 2015 and (c) high resolution images from Bing Maps. These datasets were analyzed using the open source geographic information system QGIS [46], and all of the rice fields of 2014–2015 were created by digitizing manually. The ground reference map assisted in sample selection for training the classifiers and accuracy assessment.

### 3.5. Available Agriculture Statistics Data

Agriculture statistics data for the year 2012–2013 was obtained from the Director of Economics and Statistics, Government of Assam [47]. The dataset provides the estimation of cropped area, yield and production of major crops at the district level. To validate the paddy rice map, the derived rice area was compared to the agriculture statistics data. During the time of this research, the latest government’s statistics data were not available, and, therefore, the research relied only on the 2012–2013 dataset. There were no significant interannual variabilities of temperature and rainfall between the year of 2012–2013 (statistics year) and 2014–2015 (mapping year). Therefore, the differences in the paddy rice areas in these years were attributed only to the farmer’s decisions and the local weather conditions.

## 4. Methods

### 4.1. General Overview of Procedure 

**Figure 3:**
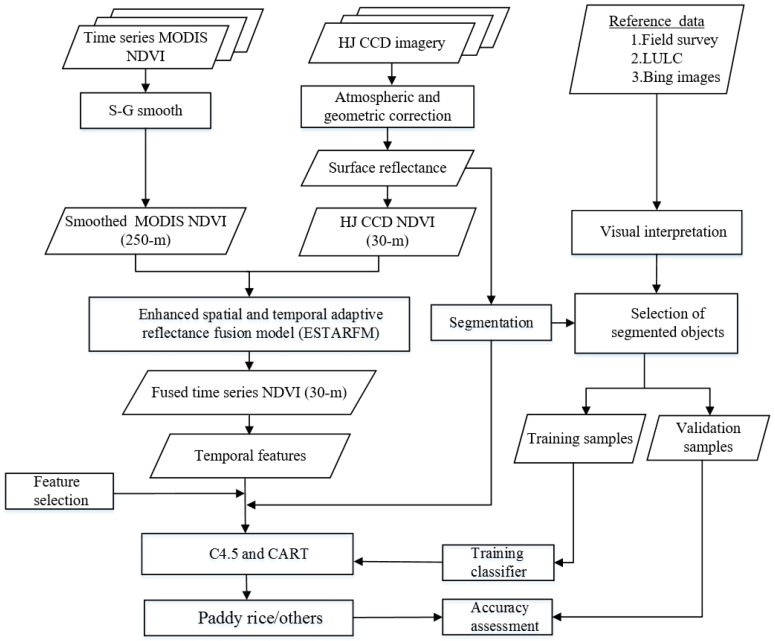
Flowchart of fine resolution paddy rice classification combining temporal features from time series coarse resolution data.

Figure 3 presents the workflow of the methodology. At first, all of the remote sensing datasets were pre-processed so that datasets are of good quality. The pre-processing includes smoothing of the time series MODIS NDVI data using the S–G filter, atmospheric and geometric correction of the HJ CCD data. Second, the MODIS NDVI dataset was fused with the HJ CCD derived NDVI data using the enhanced spatial and temporal adaptive reflectance fusion model (ESTARFM) [33]. Third, temporal features were extracted from the fused time series NDVI data. Fourth, the HJ CCD imageries were segmented for the OBIA based paddy rice classification and the temporal features were combined with the segmented images. Fifth, the segmented images were classified using the two decision tree classifiers: classification and regression tree (CART) [48] and C4.5 [49]. Finally, accuracy assessment was performed to evaluate the utility of temporal features extracted from fused time series for OBIA based paddy rice classification of fine resolution data.

### 4.2. Fusion of Time Series MODIS NDVI and HJ CCD NDVI

**Figure 4:**
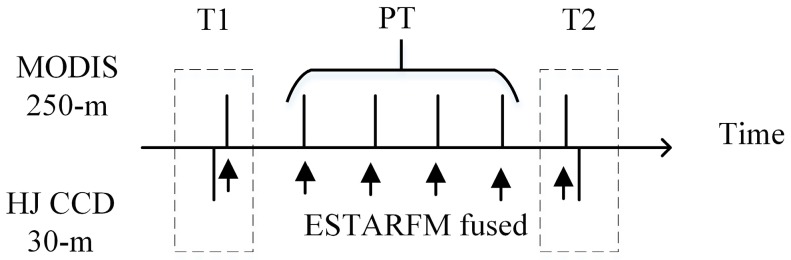
Schematic of ESTARFM fusion of MODIS NDVI and HJ CCD NDVI.

**Figure 5:**
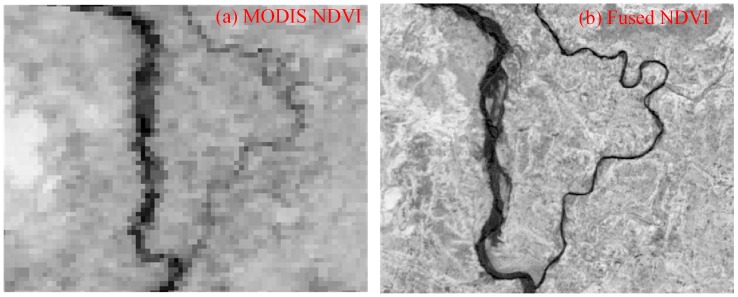
Actual and corresponding ESTARFM fused NDVI images. The fused NDVI was produced using two image pairs of HJ CCD and MODIS NDVI. (**a**) MODIS NDVI; (**b**) Fused NDVI.

ESTARFM [33] was selected to fuse the high temporal resolution MODIS NDVI and the high spatial resolution HJ CCD NDVI. ESTARFM is an enhanced spatial and temporal adaptive reflectance fusion model, originally developed for the fusion of MODIS and Landsat data for complex heterogeneous regions. The ESTARFM method calculates the pixel values based upon the spectral similarity between the fine and the coarse resolution pixels. The ESTARFM algorithm observed the reflectance trend between two time points and applied the spectral unmixing technique for better prediction of reflectance changes in heterogeneous landscape [33]. In this study, two pairs of HJ CCD and MODIS images acquired at T1 and T2 days, as well as a MODIS image on the prediction day (PT) between the date of image pairs, were used for the fusion as shown in Figure 4. The pre-processed MODIS NDVI time series dataset was reprojected and resampled to the HJ CCD resolution and extent using the MODIS Reprojection Tool (MRT), and used the two datasets as an input to the ESTARFM fusion. In the fusion process, the HJ CCD image pairs nearest to the date of MODIS images were selected in order to minimize the fusion uncertainty. The fine resolution HJ CCD images acquired at the three key growing stages (planting, heading and ripening) of paddy rice were used to capture the reflectance changes caused by phenology. The fusion generated the time series NDVI at 30-m spatial resolution at 16-day time intervals. Figure 5 shows an actual and fused NDVI image. Many previous studies demonstrated that the fusion model generates accurate synthetic images [34,50,51,52,53]; these studies also demonstrated that the fusion generated time series is usually a reasonable choice for the studies of vegetation and seasonality [34,50,51,52,53].

### 4.3. Temporal Feature Extraction and Ranking 

A set of temporal features was extracted from the fused NDVI time series data (Table 3). The temporal features characterize the seasonal photosynthetic activity and phenological patterns of plants [54]. Each crop type has distinctive temporal features. Therefore, they are helpful for crop classification. The maximum, the minimum, the mean and the standard deviation values were directly calculated from the stack of the time series. The base NDVI value (BV), the amplitude (Amp), the left derivative (LD), the right derivative (RD), the large seasonal integral (LI) and the small seasonal integral (SI) were calculated by fitting the NDVI time series to the asymmetric Gaussian function [55]. The BV is related to soil conditions, LD and RD are the greening and browning rate of vegetation, respectively, LI are related to vegetation production over the growing season and SI is an indicator of seasonally active vegetation production for a growing season.

The optimal features were evaluated and selected by the ReliefF algorithm [56] to reduce the data redundancy, inter correlation, computational complexity and classification uncertainty. The ReliefF algorithm estimates the quality of the features by ranking them according to the distances between the nearest instances, characteristics of data including the multiclass problems, incompleteness and noise [56]. The ranks of the features evaluated by ReliefF are provided in Table 4. The top ten best-ranked features were further composited with the HJ CCD spectral data for paddy rice classification. The composited dataset contains spectral information from the HJ CCD data as well as plant phenological information from the temporal features.

### 4.4. Image Segmentation for OBIA

The first step of an object-based image analysis is image segmentation. The optimal spatial units for our classification are the parcels cropped with rice. The parcels can be optimally generated as an object or segment by applying the image segmentation methods. The image segmentation method divides the image into small objects with certain homogeneity criteria and add additional spectral, spatial and textural information to it [26]. The multi-resolution segmentation [57] algorithm implemented in eCognition [27] was used for the purpose. The blue, green, red and near-infrared (NIR) bands of HJ CCD images were utilized for the segmentation. In the multi-resolution segmentation, scale parameter is a key factor for obtaining the best segmentation results. To estimate the optimum scale parameter, local variance (LV) of object heterogeneity was used as a measure [58]. The image was segmented repeatedly with multiple scales in a bottom-up manner, and the LV was calculated for each scale. Then, a graph was constructed by plotting the LV values against all of the scale parameters to investigate the changes in object heterogeneity. The thresholds in the amount of change of LV (ROC) value indicates the most appropriate scale parameter for the image.

**Figure 6:**
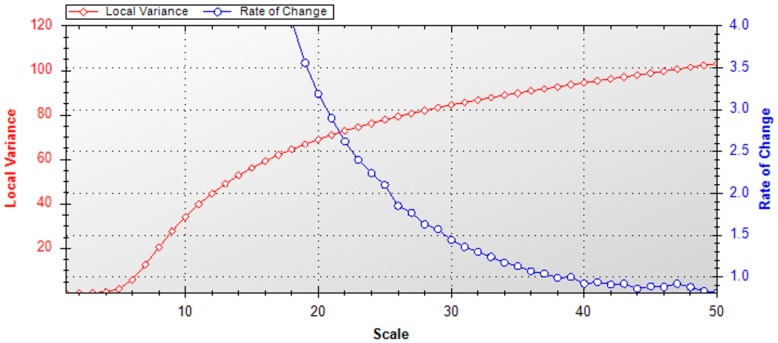
Graph of local variance (LV) and rate of change (ROC) at different scales for HJ CCD images with shape: 0.1 and compactness: 0.7.

In this study, the images were segmented at 50 different scale levels from 1 to 50 by the bottom-up approach using the multi-resolution segmentation method. The shape and compactness was kept at 0.1 and 0.7, respectively. The plotted graph between the LV and the ROC is shown in Figure 6. As noticed from the figure, the estimated scale parameter is 25, where the LV changes abruptly. Visually, the segmented objects had comparable matching with the actual field sizes. After the segmentation, the mean value of all the selected features were calculated and assigned to each object. 

### 4.5. Classification Methods

Two classification algorithms C4.5 and classification and regression tree (CART) were employed. C4.5 is a decision tree algorithm developed by Quinlan [49]. C4.5 classifies each object by constructing decision tree based on the training dataset. To construct the tree, the algorithm splits the datasets into two subsets based on the highest normalized gain, and this process repeats on each subset until all the attributes belong to the same class [59]. The algorithm was operated inside the WEKA machine learning tool [60]. During the classification, a pruning process was used using a nested 5-fold cross-validation to achieve the optimize classification accuracies by reducing over-fitting.

For comparison purposes, CART was also applied to classify the same dataset. CART is a statistical analysis based decision tree classifier widely used in remote sensing applications [61]. The tree structure of CART is determined by recursively splitting the data by threshold until ending points or terminal nodes are achieved [62]. The tree prune was conducted based on a 5-fold cross-validation process. CART was implemented using WEKA.

### 4.6. Training and Validation Sample Selection

Segmented image objects were selected as training as well as validation samples for the C4.5 and the CART classifiers. The samples were randomly collected using the ‘select samples’ tool provided in eCognition with the assistance of reference data and the knowledge of actual field situations. The sample objects were distributed uniformly representing the entire study site. A total of 200 pure samples were collected. Out of the total samples, 70% was used as training samples while the remaining 30% was used for the classification accuracy assessment.

### 4.7. Accuracy Assessment

Accuracy assessment was conducted by the confusion matrix and the kappa coefficient values [63]. The classified maps were compared to a set of randomly collected reference samples [63]. The image objects were selected as the sampling units, as this helps to assess the classification accuracy as well as the segmentation efficiency [59]. Subsequently, overall accuracy, minimum sensitivity (MS) and kappa coefficient were calculated to validate the classification results [63,64]. MS measures the accuracy of individual class objects. In this study, MS represents the accuracy of paddy rice crops. The details, discussion and justification of MS can be found in [65]. Additionally, our satellite-derived paddy areas were compared to the agriculture statistics data to assist the validation.

## 5. Results

### 5.1. Temporal Feature Analysis and Selection

Table 4 presents the rank of the top 15 features, where the larger the rank value, the more important feature is. Interestingly, five extracted temporal features were selected in the top 15, indicating the importance of temporal features in specific crop classification. The NDVI layers were selected from the two time windows: January–April and July–December. The time windows were coincided with the two paddy rice growing periods (kharif and rabi) of the region. This suggests that the phenological trajectory is crucial for differentiating paddy rice.

### 5.2. Classification Results of the Proposed and the Traditional Approaches

**Figure 7:**
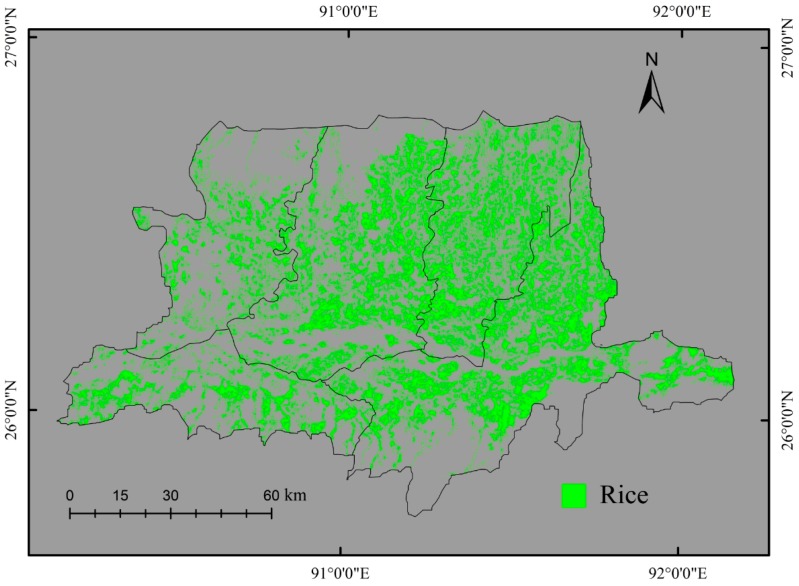
Paddy rice classification map obtained from the OI+ best-selected temporal features with the C4.5 classifier.

**Figure 8:**
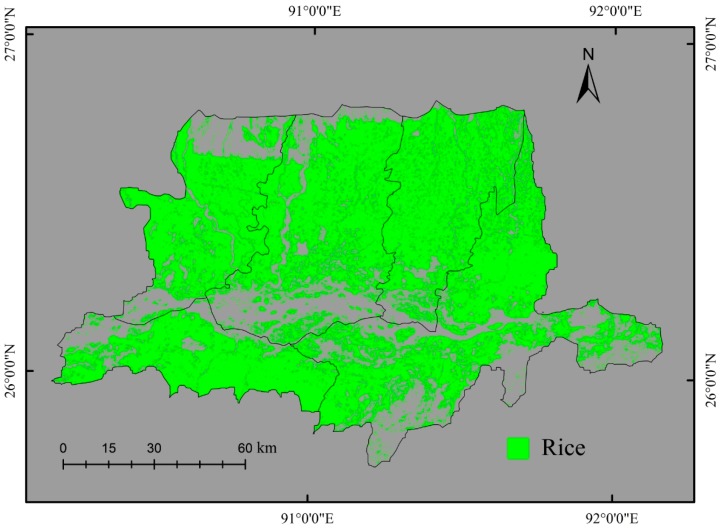
Paddy rice classification map obtained with the traditional method (i.e., the C4.5 classifier) and one HJ CCD image in the peak growing season (22 October).

The paddy rice areas were classified using the four combinations keeping the single-date October spectral image (OI) as a baseline for comparison. The combinations were: (1) (OI); (2) OI+ all temporal features; (3) OI+ best-selected temporal features; and (4) temporal spectral images. The classification was conducted using two tree based classifiers: C4.5 and CART. Classification results revealed that the paddy fields were primarily distributed in the plain areas over the region (Figure 7 and Figure 8). The classification results of combined spectral image with temporal features (combinations 2 and 3) were more accurate than using only the spectral image (combination 1). When using only the spectral image (combination 1), some objects were not classified as a paddy rice due to the similarity in spectral characteristics of paddy rice to the other land cover classes. On the other hand, when using the temporal features with the spectral image, the paddy rice was better discriminated from the other crops that have similar spectral properties to that of the paddy rice. The temporal features represented the seasonal variation of crops that helped with better classification, as seasonal characteristics differ according to crop types. Some confusion was observed in identifying paddy rice in grassland-dominated areas when using only the spectral image. However, inclusion of temporal features in the classification process removed this confusion and increased the classification accuracy. The classification of best-selected temporal features (combination 3) yielded more accurate results than using all the candidate temporal features. The feature selection strategy reduced the data redundancy and contributed to the improved results. The best classification result was obtained when multi-temporal spectral images (combination 4) were used. Although fragmented landscape impacted the classification accuracy, the presented OBIA minimized it by considering the pixel dissimilarity for the same class. 

### 5.3. Classification Accuracies

Classification accuracies of the composite images were shown in Table 5. A set of temporal features and multiple spectral images were explored in this study for paddy rice classification. As the baseline for benchmarking the temporal feature based classification, the single-date October spectral image (OI) with the C4.5 classifier yielded overall classification accuracy of 65.62% and a kappa coefficient of 0.33. The corresponding MS of the paddy rice was 57.70. Compared to the single-date OI image, the OI+ all temporal features with the C4.5 classifier achieved a significantly improved classification with the overall classification accuracy increased from 65.62% to 81.25%, and the kappa coefficient increased from 0.33 to 0.61. The classification of OI+ best-selected temporal features provided more accurate results than the single date OI and the OI+ all temporal features. The overall accuracy and kappa coefficient for the OI+ best-selected temporal features were 84.37% and 0.68, respectively. The classification of temporal spectral images outperformed all other composites when using the C4.5, yielding an overall accuracy of 90.62% and kappa coefficient of 0.81. The increment of the overall accuracies and the kappa coefficient were equal to 15.63%–18.75% and 0.28–0.35, respectively, when adding the temporal features to the OI image. In the case of the CART algorithm, the highest accuracy (87.50%) was obtained from the OI+ best-selected temporal features. For all the cases, corresponding MS was increased by the use of temporal features. The results from both the C4.5 and CART demonstrated the improvement in classification accuracies due to the use of temporal features.

### 5.4. Comparison to Available Agriculture Statistics Data

**Figure 9:**
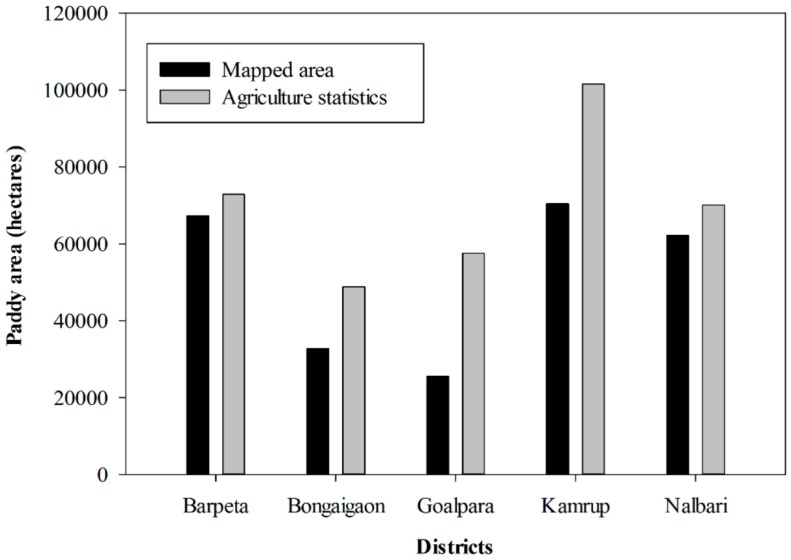
Comparison of agriculture statistics paddy rice areas of 2012 to the mapped area of 2014 for five districts.

To further validate the results, we compared the derived paddy rice areas of 2014 to the statistics reported data of 2012 to determine how accurately we captured the paddy field extent (Table 6). The derived paddy areas were from the classification results of OI+ best-selected temporal features by the C4.5 classifier. The comparison shows that satellite-derived paddy rice areas agree with the government reported statistics data, supporting the proposed approach for mapping total rice area (Figure 9). However, underestimation was observed obviously due to the comparison of different years’ datasets and the inability of identifying small paddy fields with the 30-m spatial resolution satellite data.

## 6. Discussion

Crop type discrimination using remote sensing has never been easy [66,67]. The one of the main reasons behind this is the spectral variability within the same crop due to the variable crop development schedule, influence of local weather and management practices [68]. Therefore, it is essential to consider multi-temporal observation representing crop phenology for accurate classification [67,69]. This study proposed a paddy rice classification approach that integrates temporal features from fused time series NDVI with the fine resolution spectral data. The temporal features contained the crop growth information, which represents crop phenology. Different crops have their specific phenology behavior, thus use of temporal features increase the effectiveness of paddy rice classification by providing the key crop growth stages. From the results, it was noticed that the classification uncertainties were minimized in paddy rice, grasslands and other crops when temporal features were used. Dense grasslands and some other crops have spectral properties similar to the paddy rice, but they have distinct phenology characteristics. The proposed approach showed its capability of efficient paddy rice classification in heterogeneous landscapes where several types of crops and grasslands are cohabited.

The OBIA approach proved to be robust for paddy rice classification. The OBIA considers groups of pixels for classification that minimize the variability of the same fields and lead to accurate identification of field boundaries. The OBIA showed advantages in the classification of fragmented paddy fields. The croplands are generally fragmented in the region [70]. The minimum parcel size of paddy fields in the study area was approximately 110 m × 110 m, similar to that of the statistics report [71]. This study demonstrated that OBIA based classification of HJ CCD images effectively identifies the fragmented and relatively small paddy fields.

The integration of MODIS and HJ CCD demonstrated advantages for paddy rice mapping. No single satellite sensor provides a dense time series at high spatial resolution due to the long revisit cycles and presence of cloud and snow [72]. The issue becomes more serious for rice growing regions in humid tropical climates, where cloud contamination is very frequent [73]. To increase the temporal frequency at higher spatial resolution, high temporal information MODIS was integrated with the high spatial information HJ CCD using a fusion process. This study demonstrated how to utilize a limited number of fine resolution images for the classification improvement by fusion process. The study showed that temporal features extracted from the fused dataset of MODIS and HJ CCD improved the classification of fine resolution spectral data. The launch of GF-1, Landsat 8 and Sentinel 2 has further increased the temporal frequency of 30-m spatial resolution data available for the fusion process. The proposed approach has great potential for large scale paddy rice mapping at 30-m spatial resolution integrating multi-source remote sensing data.

The 250-m MODIS NDVI time series was enhanced to 30-m spatial resolution by the ESTARFM fusion process. For the fusion, three fine resolution HJ CCD NDVI images were used as a base image. The base images covered the complete growing season of the paddy rice. Therefore, accuracy of the fused time series was not significantly decreased despite only three base images. Ideally, if more base images are used, higher fidelity is expected for the predicated finer resolution NDVI. The used base images were from the planting, heading and the ripening time of the paddy season that helped the ESTARFM to track the phenological changes in order to keep the actual trend of the fused time series. Adopting the index-then-blend [74] approach, the ESTARFM was directly applied to the NDVI time series, thereby producing a more accurate fused time series. This study demonstrated the usefulness of a few fine resolution images for the improved paddy rice classification by fusing the coarse and fine resolution data.

In this study, an approach has been demonstrated for paddy rice mapping. Generally, the paddy rice mapping algorithm based on optical images are categorized into three subgroups. The first category utilizes a single clear sky satellite image to perform statistically based unsupervised classification, supervised classification, or visual interpretation of the images in order to manually map the paddy rice areas [75,76,77]. The second category considers multi-temporal images to perform parametric or non-parametric based supervised classification [22,68]. The third category uses time series images for phenology and pixel based paddy rice mapping (PPPM) [19,31]. For the first two categories, various outcomes are expected when using varying training samples from different times or regions. It is often difficult to reuse the classifier rules and the parameters of these methods due to the existing spectral heterogeneity in different regions and time. On the other hand, the PPPM based algorithms of the third category are less affected by the aforementioned problems. The presented approach in this study belongs to the third category. The presented approach utilized the temporal features extracted from the fused NDVI time series. The temporal features represent phenology that helps make paddy rice classification more efficient. The widely used PPM based algorithm primarily requires a shortwave infrared band (SWIR) to calculate the water index, which is not available in many satellite sensors. Alternatively, the proposed approach extracts the temporal information from the time series coarse resolution images and integrated them with the fine resolution spectral features for effective mapping.

There are several potential factors that affected the results of the proposed approach. First, the temporal features are only the statistical values of time series NDVI. Other features such as start of season, end of season, and end and length of season may be included in temporal features. Second, the resolution difference between the MODIS (250-m) and the HJ CCD (30-m) may cause some uncertainties in the results. Although the MODIS images were enhanced by using fine resolution HJ CCD images, the fusion process may increase some uncertainty. Third, mixed pixels were not completely removed. Some paddy fields were very small for detection with the 30-m resolution of the HJ CCD. 

## 7. Conclusions

This study proposed an OBIA based paddy rice mapping approach that integrates temporal features of coarse resolution data with the spectral features of fine resolution data. The method’s application in northeast India revealed its efficiency, with an overall accuracy of 84.37%. The results indicated that the temporal features extracted from coarse resolution time series significantly improves the classification of the spectral data. Quantitatively, for a single spectral image, classification accuracy increased up to 18.75% from the use of temporal features. The estimated paddy areas were analogous to the agricultural statistics data with an underestimated value. The proposed approach can also be used for other crop type identification through changes in training datasets. The proposed approach has great potential in regional paddy rice mapping of fine resolution remote sensing data and more accuracy is expected, especially in homogeneous landscapes. 

Future work could be conducted on: (1) investigating the effectiveness of temporal features for fractional paddy rice mapping in order to address the sub-pixel problem; (2) investigating the texture and geometric features for paddy rice mapping and (3) investigating other classification techniques (e.g., random forest, neural networks) or other additional indicators that may enable better paddy rice classification. 

## Figures and Tables

**Table 1:** Data sets used in the study.

Satellite	Sensor	Acquisition Time (dd-mm-yyyy)	Paddy Rice Phenology Stage
HJ-1A	CCD2	22-10-2014	Heading
HJ-1A	CCD1	05-12-2014	Ripening
HJ-1B	CCD1	09-03-2015	Planting
MODIS	Terra	07-04-2014 to 22-03-2015	Sowing–Harvesting

**Table 2:** Technical specifications of HJ-1A/B.

Satellite	Sensor	Bands	Spectral Range (μm)	Spatial Resolution (m)	Swath Width (km)	Revisit Period (day)
HJ-1 A/B	CCD	1	0.43–0.52	30	360	4
		2	0.52–0.60			
		3	0.63–0.69			
		4	0.76–0.90			

**Table 3:** Feature definitions and their relationships to vegetation.

Features	Definition	Relations to Vegetation
Maximum value	The largest NDVI value of the time series	Seasonal highest greenness value
Minimum value	The smallest NDVI value of the time series	Seasonal lowest greenness value
Mean value	The mean NDVI value of the time series	Mean greenness level
Standard deviation value	The standard deviation value of NDVI time series	Standard deviation of greenness level
Base NDVI value (BV)	The average of the left and right minimum value of fitted function	Soil background conditions
Amplitude (Amp)	The difference between the maximum and the base NDVI value	Seasonal range of greenness variation
Left derivative (LD)	The ratio of the difference between the left 20% and 80% levels to the corresponding time difference	Rate of greening and vegetation growth
Right derivative (RD)	The ratio of the difference between the right 20% and 80% levels to the corresponding time difference	Rate of browning and senescence
Large seasonal integral (LI)	The sum of the representative function with a positive fit during the growing season	Vegetation production over the growing season
Small seasonal integral (SI)	The sum of the difference between the fitted function and the base level during the growing season	Seasonally active vegetation production over the growing season
23 NDVI layers	Fused time series NDVI of one year	Seasonal variation of greenness over a year

**Table 4:** Top 15 features ranked by their importance for classification.

No.	Features	Rank
1	Standard Deviation value	0.09315
2	NDVI-2015017	0.08965
3	NDVI-2015065	0.08347
4	NDVI-2014113	0.07607
5	NDVI-2014193	0.07595
6	NDVI-2014337	0.07456
7	NDVI-2014257	0.07451
8	LI	0.07302
9	Minimum value	0.07166
10	NDVI-2015001	0.07054
11	NDVI-2015081	0.07015
12	Mean value	0.07004
13	NDVI-2014209	0.07003
14	Maximum value	0.06843
15	NDVI-2014225	0.06827

**Table 5:** Classification accuracies of four composites.

Strategy	C4.5			CART		
	CCR (%)	Kappa Coefficient	Paddy Rice MS (%)	CCR (%)	Kappa Coefficient	Paddy Rice MS (%)
Single-date spectral image of October (OI)	65.62	0.33	57.70	56.25	0.14	52.40
OI+ all temporal features	81.25	0.61	90.90	81.25	0.61	90.90
OI+ best-selected temporal features	84.37	0.68	81.30	87.50	0.75	82.40
Temporal spectral images	90.62	0.81	87.50	78.12	0.55	78.60

**Table 6:** Comparison between the derived paddy rice area and the agricultural statistics. The derived paddy area corresponds to the result of OI+ best-selected temporal features with the C4.5 classifier of Figure 7.

Districts	Derived Paddy Area (2014–2015)	Agriculture Statistics (2012–2013)
	(Thousand Hectares)	(Thousand Hectares)
Barpeta	70.83	72.94
Bongaigaon	39.50	48.85
Goalpara	41.91	57.61
Kamrup	96.70	101.52
Nalbari	69.16	70.13
Total	318.10	351.05
